# Forecasting malaria in a highly endemic country using environmental and clinical predictors

**DOI:** 10.1186/s12936-015-0758-4

**Published:** 2015-06-18

**Authors:** Kate Zinszer, Ruth Kigozi, Katia Charland, Grant Dorsey, Timothy F Brewer, John S Brownstein, Moses R Kamya, David L Buckeridge

**Affiliations:** Clinical and Health Informatics Group, McGill University, 1140 Pine Ave West, Montreal, QC H3A 1A3 Canada; Children’s Hospital Informatics Program, Boston Children’s Hospital, Boston, MA USA; Uganda Malaria Surveillance Project, Kampala, Uganda; School of Medicine, University of California San Francisco, San Francisco, CA USA; University of California Los Angeles, Los Angeles, CA USA; College of Health Sciences, Makerere University, Kampala, Uganda

**Keywords:** Malaria, Uganda, Forecasting, Sentinel surveillance, Public health

## Abstract

**Background:**

Malaria thrives in poor tropical and subtropical countries where local resources are limited. Accurate disease forecasts can provide public and clinical health services with the information needed to implement targeted approaches for malaria control that make effective use of limited resources. The objective of this study was to determine the relevance of environmental and clinical predictors of malaria across different settings in Uganda.

**Methods:**

Forecasting models were based on health facility data collected by the Uganda Malaria Surveillance Project and satellite-derived rainfall, temperature, and vegetation estimates from 2006 to 2013. Facility-specific forecasting models of confirmed malaria were developed using multivariate autoregressive integrated moving average models and produced weekly forecast horizons over a 52-week forecasting period.

**Results:**

The model with the most accurate forecasts varied by site and by forecast horizon. Clinical predictors were retained in the models with the highest predictive power for all facility sites. The average error over the 52 forecasting horizons ranged from 26 to 128% whereas the cumulative burden forecast error ranged from 2 to 22%.

**Conclusions:**

Clinical data, such as drug treatment, could be used to improve the accuracy of malaria predictions in endemic settings when coupled with environmental predictors. Further exploration of malaria forecasting is necessary to improve its accuracy and value in practice, including examining other environmental and intervention predictors, including insecticide-treated nets.

**Electronic supplementary material:**

The online version of this article (doi:10.1186/s12936-015-0758-4) contains supplementary material, which is available to authorized users.

## Background

Malaria forecasting methods have become more sophisticated since Christophers’ early work [1] on forecasting malaria epidemics using rainfall, fever-related deaths, and wheat prices although the intent has remained unchanged: to inform malaria control and prevention by predicting burden or early warning of increasing burden. With the mounting cost of the global fight against malaria [2–4] and the drive towards elimination in many countries, accurate forecasts of malaria could be a valuable tool for public and clinical health services. Accurate disease predictions and early warning signals of an increase in disease burden can provide the information needed to implement targeted approaches for malaria control and prevention that make effective use of limited resources.

Malaria forecasting models have been developed in many endemic countries [5–9], although the accuracy of the models is varied and difficult to interpret across studies given the diversity of forecasting methods used, including the way in which models are evaluated [9]. Typically, these models use data on environmental risk factors, such as weather conditions, to forecast malaria for a specific geographic area over a certain interval of time. Clinical predictors, such as anti-malarial treatment, have not been explored in previous forecasting work. Inappropriate anti-malarial treatment has the potential to be a predictor of future malaria cases, for example, as it could result in the ongoing transmission of malaria within the community, leading to an increase of malaria cases at the health facility [10–12].

Uganda experiences one of the highest burdens of malaria in the world [4], where the disease is endemic in greater than 95% of the country and remains the leading cause of morbidity and mortality [13]. A national household survey in 2009 estimated a 42% malaria prevalence among children less than 5 years old [14]. Furthermore, in 2013, Uganda was ranked first in the world in terms of the number of malaria cases and ninth for malaria-related deaths [4]. The objective of this study was to determine the relevance of environmental and clinical predictors of malaria across different settings in Uganda.

## Methods

### Uganda

Uganda is an East African landlocked country situated on a large plateau, bordered by mountains, valleys, and Lake Victoria [15]. Uganda has a relatively high altitude, 1,300–1,500 m above sea level, and a mean annual temperature that ranges from 16°C in the southwest, to 30°C in the northeast and 25°C in rest of the country [14]. The vegetation is diverse, with tropical rain forests in the south, wooded savanna in central Uganda, and semi-desert conditions in the north. There are two rainy seasons in the south from March to May and from September to December, although the timing varies depending upon geographic area. In the north and northeast, there tends to be a single rainy season from April to October.

### Clinical data

**Figure 1:**
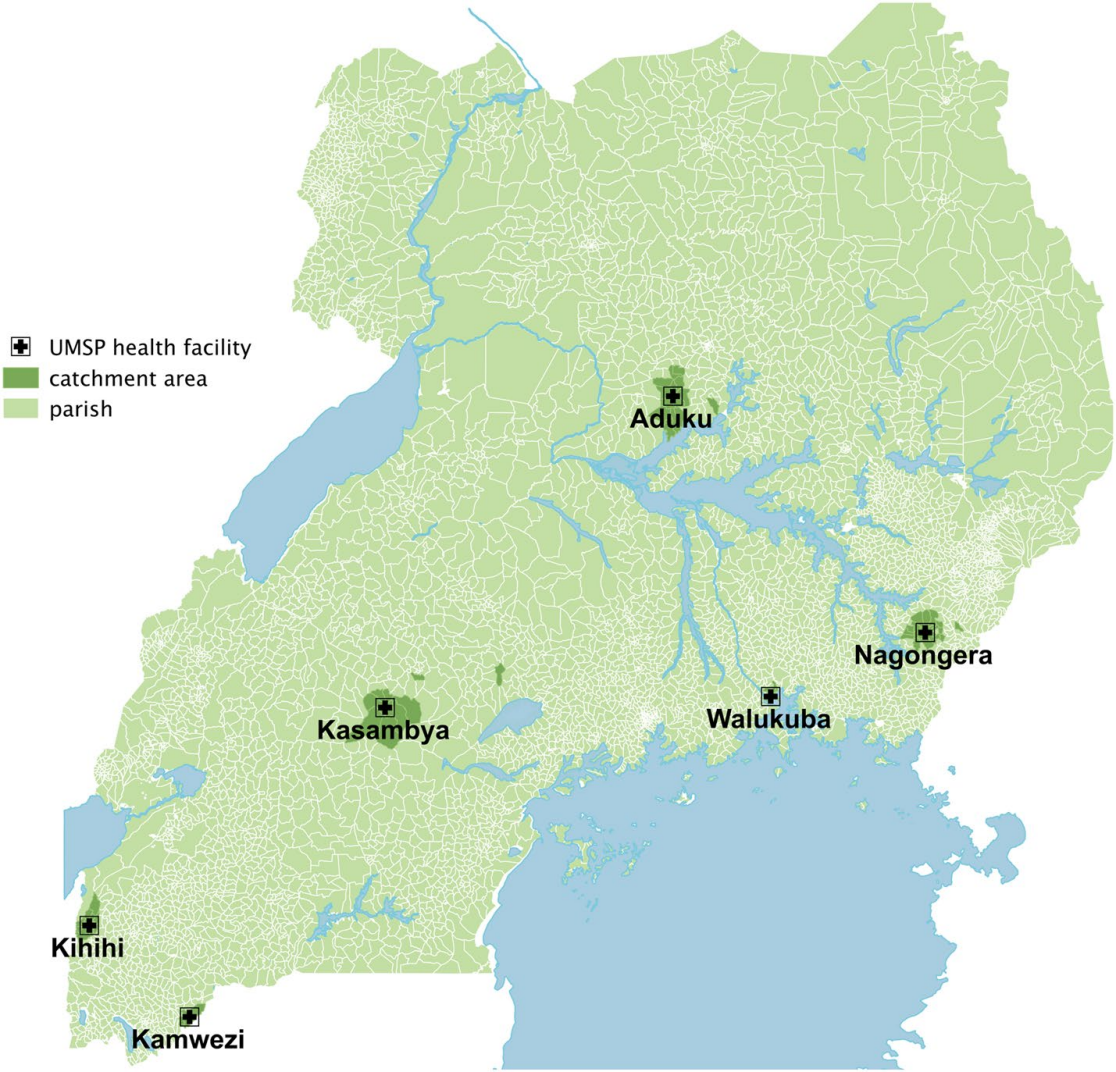
Map of the outpatient health facilities of the Uganda Malaria Surveillance Program (UMSP).

Outpatient health facility data collected by the Uganda Malaria Surveillance Project (UMSP) were used for this study. UMSP has adopted a sentinel site approach for monitoring malaria burden in Uganda. The six sentinel sites were established in pre-existing health facilities and implemented in a staggered fashion, starting in July 2006 with the last site opening in August 2008 (Figure 1). These sites were selected to represent the diversity of malaria transmission in Uganda and include six different health facilities that provide patient care free-of-charge, including diagnostic tests and medications [16]. All sentinel site staff received training in malaria diagnosis, case management, and data collection along with support for laboratory testing. Individual-level data collected from all patients presenting to the outpatient department include results of malaria diagnostic testing, diagnoses, treatments, as well as demographic information and parish of residence. Parishes are the second smallest administrative unit in Uganda with approximately 5,000–6,000 inhabitants. Data on the parish of residence for patients were used to determine the catchment area of each sentinel site [17]. The methodology for catchment definition has previously been explained but briefly, parishes of individuals with confirmed malaria were excluded from a facility’s catchment area if the observed utilization of malaria-related services was statistically significantly lower than that of the expected utilization. The number of parishes within each health facility’s catchment area ranges from three to seventeen with an average catchment population of 60,000.

### Ethical issues

Ethical approval of the study was provided by the institutional review boards at McGill University, Makerere University, and Uganda National Council for Science and Technology.

### Environmental data

Satellite sensor-derived environmental data were obtained from the Tropical Rainfall Measuring Mission (TRMM) and moderate resolution imaging spectroradiometer (MODIS) instruments onboard the Terra satellite. The TRMM product (TRMM3B42) provided daily rainfall estimates with a spatial resolution of 0.25° × 0.25° or 27.8 km × 27.8 km (at the equator). Daytime and nighttime temperature estimates (land surface temperature; LST) were obtained from MODIS (MOD11A2) using 8-day composite images at a 1 km × 1 km resolution. The enhanced vegetation index (EVI) was also processed from MODIS (MOD13A1) using 16-day composite images at a 0.5 km × 0.5 km resolution.

### Measurement

The primary outcome of interest was defined as the weekly number of laboratory-confirmed malaria cases diagnosed at each sentinel site, which are also called the response series. The predictive power of the clinical variables was assessed and included the proportion of suspected cases (defined by the presence of fever) that were tested for malaria, the number of suspected cases that tested negative for malaria, the type of anti-malarial treatment prescribed, and the appropriateness of treatment as defined by the Ugandan National Malaria Control Programme Policy [13, 18]. For example, uncomplicated malaria (laboratory confirmed) for individuals (over 5 kg, over 4 months of age, or not in the first trimester of pregnancy) should receive a prescription of an artemisinin-based combination therapy, such as artemether-lumefantrine or artesunate plus amodiaquine. For uncomplicated malaria (laboratory confirmed) for women in their first trimester of pregnancy or for infants under 5 kg or under the age of 4 months, oral quinine should be prescribed. Each series was based upon the health facility’s catchment area and the individual-level data were aggregated to a weekly frequency.

Mean EVI and mean daytime and nighttime temperature for each catchment area was calculated by overlying the parish boundaries with the raster environmental data (i.e., TRMM and MODIS). The weighted mean pixel or cell value for temperature and EVI for each parish was calculated and based on the proportion of the parish area contained within each pixel [19]. Rainfall for a parish was calculated from the pixel that intersected with the center point of the parish polygon as the TRMM pixels were often larger than a parish. Total rainfall was the cumulative total of rainfall over a 1-week period and other measures of rainfall were explored including logarithmically transformed total rainfall, maximum weekly rainfall, minimum weekly rainfall, and the weekly rainfall range. EVI and temperature values were interpolated to a weekly temporal resolution, given the different temporal frequencies. A linear spline was used to interpolate EVI and a quadratic spline to interpolate temperature measures. Approximately 9% of the observations were missing for nighttime temperature, and these values were imputed during the interpolation process using a quadratic spline. All polygons (parishes) were projected using the Universal Transverse Mercator system; zone 35 north (UTM35N).

Once weekly time series of environmental predictors were created for each parish, a weekly average across all parishes within a sentinel site’s catchment area was then calculated as a summary measure for each health facility. All environmental and clinical predictor series began from the start of consistent data collection, often coinciding with the sentinel surveillance programme’s implementation date, at each site until 31 May 2013. All series are listed in Table 1.

**Table 1:** Response series (confirmed malaria) and potential clinical and environmental predictors series

Series	Description
Clinical data	
Confirmed malaria	Number of individuals with positive microscopy or rapid diagnostic test of malaria
Negative for malaria	Number of suspected (presence of fever) tested negative for malaria
Proportion tested	Proportion of suspected (presence of malaria) tested for malaria
Appropriate treatment^a^	Number of individuals who received appropriate anti-malarial prescriptions based upon their malaria status and NMCP treatment guidelines
Artemisinin-based combination therapy (ACT)	Number of ACTs prescriptions
Appropriate ACT^a^	Number of individuals who were appropriately prescribed ACTs according to guidelines and malaria status
Quinine	Number of quinine prescriptions
Appropriate quinine^a^	Number of individuals who were appropriately prescribed quinine according to guidelines and malaria status
Chloroquine	Number of chloroquine prescriptions
Inappropriate chloroquine	Number of individuals who were prescribed chloroquine
Environmental data	
Daytime temperature	Temperature at 3 pm (8-day composite image)
Nighttime temperature	Temperature at 3 am (8-day composite image)
Total rainfall	Cumulative sum of daily rainfall over a week period
Log total rainfall	Log of cumulative sum of daily rainfall over a week period
Mean rainfall	Mean daily rainfall over a week period
Minimum rainfall	Minimum daily rainfall over a week period
Maximum rainfall	Maximum daily rainfall over a week period
Rainfall range	Difference between the maximum and minimum rainfall over a week period
Vegetation	Enhanced vegetation index (16-day composite image)

^a^Inappropriate treatment was also a potential predictor which was the opposite of appropriate treatment (e.g., the number of individuals who were not prescribed an ACT when they should have or were prescribed inappropriately).

### Analysis

ARIMA (autoregressive integrated moving average) models are regressions that are designed to account for serial autocorrelation in time series [20]. The ARIMAX form of ARIMA models was used for this study, which is a multivariate autoregressive integrated moving average model and includes current and past values of independent variables as predictors. The ARIMAX (*p,d,q*) models were fitted with three components: the autoregressive order (*p*), differencing order (*d*), and the moving average order (*q*). ARIMAX models were developed for each sentinel site with a 52-week forecast horizon, resulting in 52 weekly forecasts over the year period of 1 June 2012–31 May 2013. The series were divided into training (site implementation-31 May 2012) and testing (1 June 2012–31 May 2013) series. The training portion of the series was used for model building and the testing series were reserved for assessing the forecast accuracy of the models.

The best possible combination of environmental and clinical covariates was determined by Akaike’s information criterion (AIC) using the training series. The fit of the training series model was reassessed using the AIC-value, after it was adjusted for the model parameters. Following pre-whitening, the significant lags between the predictor series and the response series were identified and considered in the model building. This would allow the lag between, for example, a rainfall event and the associated response in the malaria series, to be incorporated into the model. The cross-correlations of predictor and response series (pre-whitened series) were assessed to determine significant lags [21].

Model fit was assessed through inspection of residual autocorrelation diagnostics via the autocorrelation function (ACF), the partial autocorrelation function (PACF), and the Ljung–Box test. Further, histograms of the residuals and normal quantile plots were used to assess the distribution of the residuals. The model fit and model building was an iterative process. Each model was used to generate weekly forecasts up to 52 weeks in the future, from 1 June 2012 to 31 May 2013, using the parameters for predictors that were chosen during model selection. The models were implemented in a rolling fashion; after each weekly forecast, the model would be updated to include the observed malaria counts and covariate values that occurred during that week before forecasting the malaria counts for the following week.

The final model for each site analysed for accuracy, by comparing the actual versus forecasted cases for the testing period (1 June 2012 until 31 May 2013) for each week or forecast horizon during the 52 weeks of forecasting. The symmetric mean absolute percentage error (SMAPE) was used to measure accuracy:

$${\text{SMAPE}}aveh = \frac{1}{n}\mathop \sum \limits_{i = 1}^{n} \frac{{\left| {Y_{i} - \hat{Y}_{i} } \right|}}{{{{(Y_{i} + \hat{Y}_{i} )} \mathord{\left/ {\vphantom {{(Y_{i} + \hat{Y}_{i} )} 2}} \right. \kern-0pt} 2}}} \times 100$$

where SMAPE_*aveh*_ is the average SMAPE value for a horizon (*h*), *Y*_*i*_ is the observed value, $\hat{Y}_{i}$ is the forecasted value, and *n* is the number of forecasts or observations for that horizon [22]. There were multiple forecasts per forecast horizon, given the rolling implementation of the models. Therefore, a SMAPE value was calculated for each horizon, resulting in a total of 52 SMAPE values, one for each forecast horizon. For example, there were 52 forecasts of all 1-week ahead forecasts (horizon one) and the error of these forecasts would be averaged to obtain an overall one-week ahead forecast error. Negative forecast values were set to zero. The upper bound of the SMAPE metric is 200%, producing a range of possible values from 0 to 200%. In addition, the percent error of the total forecasted burden (number of cases) over the 52-week period was compared to the actual burden that occurred from 1 June 2012 to 31 May 2013. The first forecasted observation from each forecast horizon (1 to 52) was used to obtain the cumulative forecasted burden over the forecasting period.

RStudio v0.93 was used for data management, SAS v9.3 was used for the analyses, ERDAS Imagine v10.1 was used for processing the satellite images, and ArcGIS v10 was used for spatial analyses.

## Results

There were slightly more females than males with confirmed malaria at each site and the mean annual temperatures were relatively similar with a 2°C difference across the sites (Table 2). Nagongera had the youngest malaria cases in age and generally, there were slightly more female than male confirmed malaria cases at each site. On average, Nagongera had the highest daytime temperatures (29.8°C) and received the most rain in 2012 (1.66 m).

**Table 2:** Characteristics for each UMSP outpatient health facility

Site	Series start (no. of weeks)^a^	Cumulative number of cases	Average age (years)	% Female	Average daytime temperature (°C)	Average nighttime temperature (°C)	Cumulative rainfall for 2012 (m)
Aduku	5 November 2007 (291)	14,963	10.7	59	29.1	17.6	1.29
Kamwezi	8 September 2008 (247)	18,882	15.8	56	27.7	15.8	1.01
Kasambya	10 March 2008 (273)	20,636	13.7	57	27.3	15.4	1.02
Kihihi	9 June 2008 (261)	21,278	20.0	63	27.3	16.5	1.05
Nagongera	16 June 2008 (259)	20,716	8.4	56	29.8	17.5	1.66
Walukuba	28 April 2008 (266)	29,664	15.0	59	28.6	16.9	1.22

^a^Total number of weeks or time points for the series (training and testing series).

The predictors included in the final models varied by sentinel site (Table 3). A commonly included category of predictor was drug treatment, with at least one treatment predictor series included in every model. Appropriate treatment and the number of courses of ACT were the most frequently included treatment predictors. Total rainfall was the most commonly retained environmental predictor. Kamwezi’s models contained the smallest number of predictors, four, whereas Walukuba contained the most, fourteen. Approximately half the predictor series were lagged, ranging from lags of 1 to 52 weeks. A table of the predictors, parameters, and lags are included in an appendix (See Additional file 1: Table S1).

**Table 3:** Categories of clinical and environmental predictors included in final forecasting models

Predictor	Aduku	Kamwezi	Kasambya	Kihihi	Nagongera	Walukuba
Rainfall	✓	✓	✓	✓	✓	✓
Temperature	✓		✓	✓	✓	✓
Vegetation	✓	✓	✓	✓	✓	✓
Treatment	✓	✓		✓	✓	✓
Suspected	✓	✓	✓		✓	✓
Proportion screened	✓		✓	✓	✓	✓

Seasonality was visually assessed for each model using the ACF and PACF, although none of the models included seasonal terms as non-seasonal parameters and predictors were sufficient to capture any seasonal variation. All models had one order of differencing and a moving average term of one order. However, the orders of the autoregressive (AR) terms ranged from 1 to 43.

**Figure 2:**
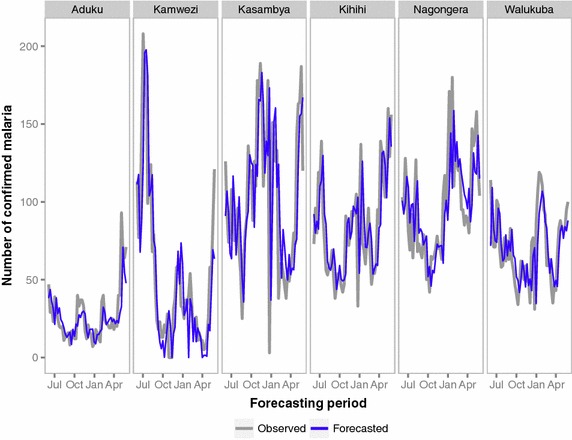
Plot of weekly observed and forecasted malaria counts (horizon 1 forecasts) for each UMSP site from 1 June 2012 to 31 May 2013.

**Figure 3:**
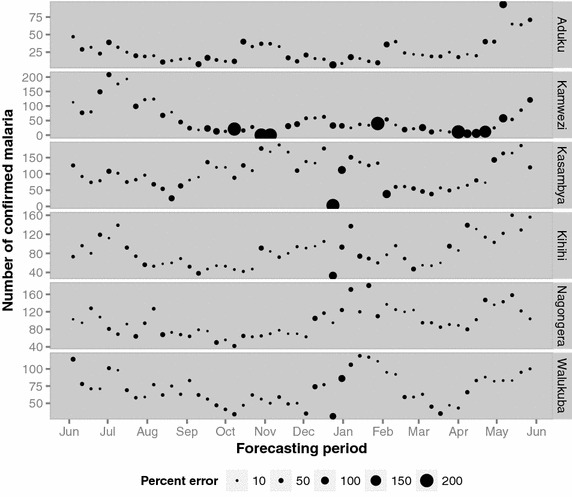
Plot of weekly-observed malaria counts sized by SMAPE error for forecast horizon 1 for each UMSP site from 1 June 2012 to 31 May 2013. Each weekly observed count of malaria is sized proportionally to the forecasting error associated with that particular observation.

**Figure 4:**
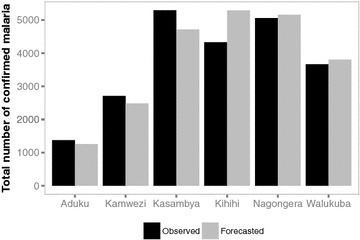
Bar chart of the total observed malaria burden versus burden prediction by UMSP site from 1 June 2012 to 31 May 2013.

The short-term horizons (e.g., 1- to 4-weeks ahead) were better at predicting high-frequency variation in malaria cases compared to longer-term horizons although the short-term horizons predicted the peaks 1 to 4 weeks after they were observed (Figure 2). Kamwezi had the highest error with an average of 128% across all 52 forecast horizons and Nagongera had the lowest average error at 27% (Table 4). When examining the forecasting accuracy by forecast horizon, horizon one forecasts (i.e., 1-week ahead forecasts), typically resulted in the smallest error. The weekly SMAPE error was highest when the observed counts were low or zero which occurred most often with the Kamwezi site (Figure 3). When examining the ability of models to predict the total number of cases during the forecasting period, Nagongera had the lowest percent error at 2% (Figure 4). The Kihihi model had the largest error with an overprediction of 22% and the average error was 9% for the 52-week forecasted malaria burden across all six sites.

**Table 4:** Error for selected forecast horizons by UMSP site

Site	Horizon 1 (%)	Horizon 4 (%)	Horizon 12 (%)	Horizon 26 (%)	Horizon 52 (%)	Average (%)^a^
Aduku	31.6	43.2	62.1	73.7	99.5	70.7
Kamwezi	57.8	117.0	125.6	147.1	127.0	127.8
Kasambya	31.3	42.8	56.0	42.9	13.5	46.6
Kihihi	20.9	31.1	46.3	31.2	33.4	37.0
Nagongera	19.4	27.8	32.5	31.9	2.0	26.3
Walukuba	22.1	30.7	35.2	37.3	34.6	30.8

^a^Average error across all forecast horizons (horizons 1–52).

## Discussion

The Abuja Declaration noted the importance of accurate disease prediction for targeting and evaluating control measures [23]. For forecasting models to be useful for clinical and public health decision-making, models must produce accurate forecasts. This study examined various predictors across six different settings in Uganda and consistently found that both environmental and clinical predictors were necessary to achieve the highest possible predictive power. This is the first study that examines clinical predictors, other than malaria cases, in combination with environmental predictors for forecasting malaria. Future forecasting work should consider clinical predictors given the likelihood of their relevance in different endemic settings.

Incorporating clinical predictors such as anti-malarial treatment, the proportion of individuals screened for malaria, and the number of malaria negative individuals, produced models with the best predictive power across a range of settings in Uganda and across forecast horizons. In addition, rainfall, temperature, and EVI were also identified as necessary for several of the models in terms of achieving the greatest predictive ability. The accuracy of the models varied widely between the sites, with models at some sites (e.g., Kamwezi) influenced by low and zero counts in the response series, leading to large relative error measures (200%).

It is not known if the observed cases were incident or recrudescent. Inclusion of recrudescent cases in the outcome series would weaken the predictive ability of environmental covariates [5], which have a stronger relationship with incident cases, although inclusion of recrudescent cases may strengthen the predictive ability of certain treatment predictors [10–12]. There are different ways in which measurement error could have influenced the findings. Remote sensing data was used in lieu of ground observations due to data availability, and these remote sensing observations are subject to measurement error [23–29]. The treatment data were based upon prescriptions and not on dispensed anti-malarial medication or treatment taken by the patient, which may have introduced noise into the series, and facility-level factors likely influenced the accuracy of the observed counts of confirmed malaria. Finally, incorporate other predictors, such as humidity and intervention data (e.g., insecticide-treated nets, indoor residual spraying), were not included which may further improve the forecasting accuracy. The Aduku region, for example, has been subject to rounds of indoor residual spraying [30], which likely accounts for some of the unexplained variation. All of these factors have likely resulted in measurement error, increasing the noise of the different series and decreasing their ability to predict malaria.

The models were not developed to explain causal relationships but were developed with the goal of achieving the highest predictive power. Consequently, multicollinearity was present between various predictor series and influenced which predictors and respective lags were included in the final models. The biological interpretation of specific lags and combination of predictors is therefore limited.

There are different potential users of malaria forecasts. Health facilities could use the forecasts to plan for patient visits, for example, in ensuring that sufficient diagnostic and treatment materials are available. Policy-makers and those involved with malaria control strategy planning could use the information to understand the burden of malaria in a particular location for the coming year, to inform the procurement of anti-malarials and diagnostic equipment, and also in informing malaria control strategy, such as targeting intervention efforts. With the increasing availability of electronic medical records and electronic systems, clinical predictors could be collected and analysed in real-time in conjunction with remote sensing data, if meteorological data are not an option. Using malaria forecasting models in practice would also allow us to understand how accurate a model needs to be, in order to be useful. Potential barriers to the utility of the models include the supply chain management approach, if supply decisions are made at the national level through a national store (‘push’ system) [31] versus at the health-facility level as well as a lack of resources required to guide community-tailored prevention measures.

## Conclusions

Clinical data such as drug treatment could be used to improve the accuracy of malaria predictions in a highly endemic setting when coupled with environmental predictors. Future research should consider other non-environmental predictor series and the practical implications of accuracy should be examined to determine the impact of forecast accuracy on disease control decisions. Accurate malaria forecasting models are needed to guide efficient allocation of resources for prevention and response; further exploration of malaria forecasting is necessary to improve its value in practice.

## Additional file

Additional file 1:
** Table S1**. Description of final models. Table describing the final ARIMA(p,d,q) model, predictors, and associated lags.

## Abbreviations

AIC: Akaike’s information criterion
ACF: autocorrelation function
ACT: artemisinin-based combination therapy
AR: autoregressive
ARIMA: autoregressive integrated moving average
EVI: enhanced vegetation index
MODIS: moderate resolution imaging spectroradiometer
PACF: partial autocorrelation function
SMAPE: symmetric mean absolute percentage error
TRMM: Tropical Rainfall Measuring Mission
UMSP: Uganda Malaria Surveillance Project
UTM35N: Universal Transverse Mercator system zone 35 north
