# Supplementary material for: Forecasting malaria in a highly endemic country using environmental and clinical predictors
Source: Malar J. 2015 Jun 18;14:245. doi: 10.1186/s12936-015-0758-4 (PMC4470343; doi:10.1186/s12936-015-0758-4)
Supplement: Additional file 1: — Table S1. Description of final models. Table describing the final ARIMA(p,d,q) model, predictors, and associated lags. [file 12936_2015_758_MOESM1_ESM.docx]

## Additional file

## Table S1 Description of final models

| **Site** | **ARIMA(p,d,q)** | **Predictor (lag)** |
| --- | --- | --- |
| Aduku | (43,1,1) | log total rainfall, total rainfall (26), daytime temperature (50), nighttime temperature (8), proportion tested (1), negative for malaria (10), appropriate treatment, appropriate ACTs |
| Kamwezi | (19,1,1) | total rainfall (34), negative for malaria, ACTs, inappropriate chloroquine |
| Kasambya | (7,1,1) | log total rainfall, daytime temperature (42), vegetation (36), negative for malaria |
| Kihihi | (4,1,1) | total rainfall (43), rainfall range (4), maximum rainfall (18), daytime temperature (12), nighttime temperature, proportion tested (52), appropriate treatment, ACTs, inappropriate quinine, chloroquine |
| Nagongera | (34,1,1) | log total rainfall, rainfall range, nighttime temperature (10), proportion tested, appropriate treatment, appropriate ACTs |
| Walukuba | (13,1,1) | daytime temperature (15), vegetation, proportion tested, negative for malaria, appropriate treatment, inappropriate treatment (52), ACTs, appropriate ACTs, quinine (43), appropriate quinine, chloroquine (43), inappropriate chloroquine (39) |
